# Draft Genome Sequences of Two Dothideomycetes Strains, NU30 and NU200, Derived from the Marine Environment around Sugashima, Japan

**DOI:** 10.1128/mra.01217-22

**Published:** 2023-04-17

**Authors:** Gakuho Kurita, Gohta Goshima, Kazuma Uesaka

**Affiliations:** a Sugashima Marine Biological Laboratory, Graduate School of Science, Nagoya University, Toba, Japan; b Department of Biological Science, Graduate School of Science, Nagoya University, Nagoya, Japan; c Centre for Gene Research, Nagoya University, Nagoya, Japan; University of California, Riverside

## Abstract

Several marine-derived black yeast species belonging to the class Dothideomycetes grow and divide in an unconventional manner, thereby attracting the interest of cell biologists. Here, we report the draft genome sequences of two black yeast strains, which comprised 25.5 Mb and 27.7 Mb and 172 and 65 contigs, respectively.

## ANNOUNCEMENT

**FIG 1:**
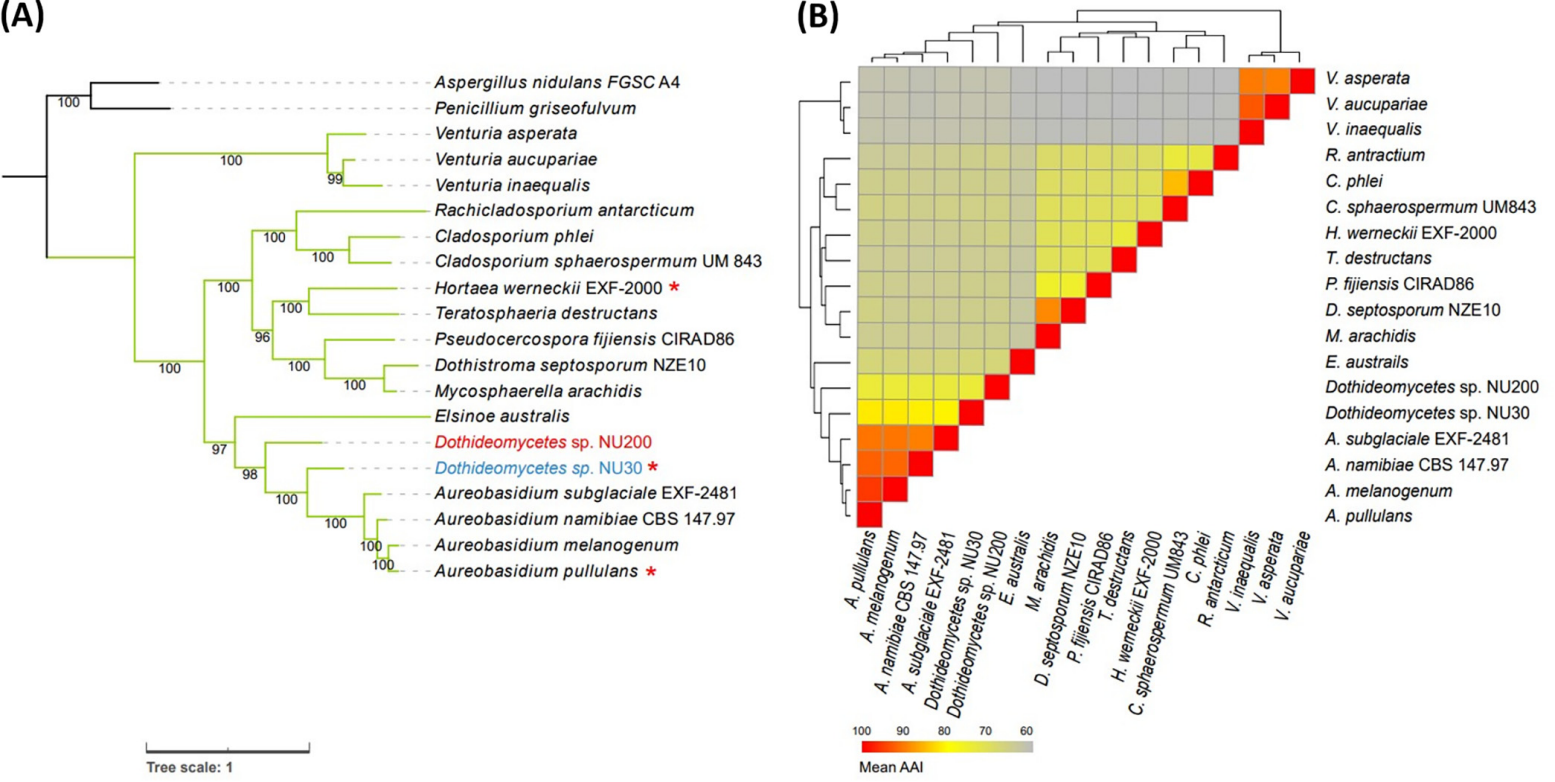
Phylogenetic relationships of species within the class Dothideomycetes. (A) Phylogenetic reconstruction of representative genomes in the class Dothideomycetes. The tree was constructed using the maximum likelihood (ML) method based on concatenated amino acid sequences of RPB2, α-tubulin, TIF5, and TOP1. Branch labels represent the bootstrap values. Green, Dothideomycetes; *, species that show cell density-dependent growth/division mode alteration (3). (B) Heat map representing the pairwise average amino acid identity (AAI) in the class Dothideomycetes. Protein sequences were obtained from BUSCO complete single-copy proteins.

Dothideomycetes is a highly diverse class in the phylum Ascomycota, inhabiting various niches, including marine environments (1). Several black yeast species in Dothideomycetes alternate their modes of growth and division in laboratory cultures (2, 3). Strain NU30 changes its growth and division modes depending on the cell density (3). NU30 and another black yeast, NU200, were isolated from a marine environment at the Marine Biological Laboratory, Nagoya University, on the island of Sugashima (Toba, Japan; NU30, April 2020; NU200, October 2021). Seawater from an outdoor tank, in which various marine animals and seaweeds were present (see Fig. 1 in reference 3), was plated onto a yeast extract-peptone-dextrose (YPD) agar plate containing antibiotics (20 μg/mL carbenicillin, 100 μg/mL chloramphenicol, and 10 μg/mL tetracycline) (3). After incubation at 25°C, the fungal colonies were transferred onto fresh YPD medium to obtain clone cultures. NU30 and NU200 were chosen for whole-genome sequencing because their phylogenetic positions could not be determined using DNA barcode sequencing.

Genomic DNA was extracted from NU30 and NU200 cells grown exponentially in YPD liquid medium for 3 days using a Dr. GenTLE kit (TaKaRa Bio). After confirming the purity of the extracted DNA (optical density at 260/280 nm [OD_260/280_], 1.77 and 1.93 for NU30 and NU200, respectively), DNA libraries were prepared (at Novogene) using a NEBNext Ultra II DNA library prep kit. Sequencing was performed using an Illumina NovaSeq 6000 system, and 2 × 150-nucleotide paired-end reads were generated (NU30, 15,378,531; NU200, 13,432,064). The read quality was checked using FastQC v0.11.9 (4) and fastp v0.23.2 (5). *De novo* assembly was performed using SPAdes v3.13.0 (6). Assembly errors were polished twice using Pilon v1.24 (7) for each strain. The organelle genome was assembled separately using GetOrganelle v1.7.6.1 (8). The genome assembly of strain NU30 resulted in 172 contigs with a total length of 25.5 Mb (*N*_50_, 271,977 bp; G+C content, 48.95%), with a complete mitochondrial genome length of 41,311 bp, whereas the assembly of strain NU200 was 27.7 Mb long and consisted of 65 contigs (*N*_50_, 872,493 bp; G+C content, 49.36%), with a complete mitochondrial genome length of 34,404 bp. Analysis of the genome completeness, conducted using Benchmarking Universal Single-Copy Orthologs (BUSCO) v5.3.2 (9) with the dothideomycetes_odb10 data set, resulted in values of 96.6% and 95.9% for NU30 and NU200, respectively. Unless otherwise stated, default parameters were used for the analysis.

Concatenated amino acid sequences of DNA-directed RNA polymerase II core subunit (RPB2), α-tubulin, eukaryotic translation initiation factor 5 (TIF5), and DNA topoisomerase I (TOP1) were used to construct a phylogenetic tree. These protein sequences were obtained from single-copy orthologs of BUSCO output (8) and manually checked for completeness. A multiple sequence alignment was constructed using MAFFT v7.505 (10) and trimmed using ClipKIT v1.3.0 (11) with the “kpi” option. The aligned sequences were manually concatenated. The ML-based tree inference was performed using IQ-TREE v2.2.0.3 (12) with the option “-m MFP -bb 1000 -alrt 1000.” Penicillium griseofulvum and Aspergillus nidulans FGSC A4 were used as outgroups. The tree suggested that NU30 and NU200 were both included in the class Dothideomycetes (Fig. 1A). Pairwise average amino acid identity (AAI) calculation using BUSCO complete proteins were consistent with the phylogenetic tree and indicated that NU200 and NU30 were distant from known species (Fig. 1B). CompareM v0.1.2 (https://github.com/dparks1134/CompareM) was used to calculate the AAI. The pheatmap v1.0.12 package (13) was used for hierarchical clustering of the AAI distance matrix and the subsequent heat map visualization (with the “dist.method = euclidean,” and “clustering_method = mcquitty” options). Cell density-dependent alteration in the growth/division modes has been observed for NU30, Hortaea werneckii, and Aureobasidium pullulans, but not for *Cladosporium* spp. (2, 3) (Fig. 1A). Thus, diverse, but not all, genera in Dothideomycetes are endowed with the plastic nature of cell proliferation. The two draft genome sequences presented here enrich the list of Dothideomycetes genomic information, building a foundation for deeper understanding of their biology.

### Data availability.

The raw sequencing reads were deposited at the DNA Data Bank of Japan (DDBJ)/GenBank under BioProject accession number PRJDB14294, BioSample accession numbers SAMD00529716 and SAMD00529717, and SRA accession numbers DRX392176 and DRX392177. The draft genome sequences of Dothideomycetes sp. strain NU30 and Dothideomycetes sp. strain NU200 were deposited at DDBJ/ENA/GenBank under accession numbers BSBH01000001 to BSBH01000172 and BSCJ01000001 to BSCJ01000065, respectively. The complete mitochondrial genome sequences of NU30 and NU200 were deposited individually at DDBJ/ENA/GenBank under accession numbers LC735278 and LC739045, respectively.

## References

[1] Pem D, Jeewon R, Chethana KWT, Hongsanan S, Doilom M, Suwannarach N, Hyde KD. 2021. Species concepts of Dothideomycetes: classification, phylogenetic inconsistencies and taxonomic standardization. Fungal Divers 109:283–319. doi:10.1007/s13225-021-00485-7.

[2] Mitchison-Field LMY, Vargas-Muñiz JM, Stormo BM, Vogt EJD, Van Dierdonck S, Pelletier JF, Ehrlich C, Lew DJ, Field CM, Gladfelter AS. 2019. Unconventional cell division cycles from marine-derived yeasts. Curr Biol 29:3439–3456.e5. doi:10.1016/j.cub.2019.08.050. 31607535 PMC7076734

[3] Goshima G. 2022. Growth and division mode plasticity is dependent on cell density in marine-derived black yeasts. Genes Cells 27:124–137. doi:10.1111/gtc.12916. 34932251

[4] Andrews S. 2010. FastQC: a quality-control tool for high-throughput sequence data. Babraham Institute, Cambridge, UK.

[5] Chen S, Zhou Y, Chen Y, Gu J. 2018. fastp: an ultra-fast all-in-one FASTQ preprocessor. Bioinformatics 34:i884–i890. doi:10.1093/bioinformatics/bty560. 30423086 PMC6129281

[6] Bankevich A, Nurk S, Antipov D, Gurevich AA, Dvorkin M, Kulikov AS, Lesin VM, Nikolenko SI, Pham S, Prjibelski AD, Pyshkin AV, Sirotkin AV, Vyahhi N, Tesler G, Alekseyev MA, Pevzner PA. 2012. SPAdes: a new genome assembly algorithm and its applications to single-cell sequencing. J Comput Biol 19:455–477. doi:10.1089/cmb.2012.0021. 22506599 PMC3342519

[7] Walker BJ, Abeel T, Shea T, Priest M, Abouelliel A, Sakthikumar S, Cuomo CA, Zeng Q, Wortman J, Young SK, Earl AM. 2014. Pilon: an integrated tool for comprehensive microbial variant detection and genome assembly improvement. PLoS One 9:e112963. doi:10.1371/journal.pone.0112963. 25409509 PMC4237348

[8] Jin J-J, Yu W-B, Yang J-B, Song Y, dePamphilis CW, Yi T-S, Li D-Z. 2020. GetOrganelle: a fast and versatile toolkit for accurate de novo assembly of organelle genomes. Genome Biol 21:241. doi:10.1186/s13059-020-02154-5. 32912315 PMC7488116

[9] Simão FA, Waterhouse RM, Ioannidis P, Kriventseva EV, Zdobnov EM. 2015. BUSCO: assessing genome assembly and annotation completeness with single-copy orthologs. Bioinformatics 31:3210–3212. doi:10.1093/bioinformatics/btv351. 26059717

[10] Katoh K, Misawa K, Kuma K-I, Miyata T. 2002. MAFFT: a novel method for rapid multiple sequence alignment based on fast Fourier transform. Nucleic Acids Res 30:3059–3066. doi:10.1093/nar/gkf436. 12136088 PMC135756

[11] Steenwyk JL, Buida TJ, Li Y, Shen X-X, Rokas A. 2020. ClipKIT: a multiple sequence alignment trimming software for accurate phylogenomic inference. PLoS Biol 18:e3001007. doi:10.1371/journal.pbio.3001007. 33264284 PMC7735675

[12] Minh BQ, Schmidt HA, Chernomor O, Schrempf D, Woodhams MD, von Haeseler A, Lanfear R. 2020. IQ-TREE 2: new models and efficient methods for phylogenetic inference in the genomic era. Mol Biol Evol 37:1530–1534. doi:10.1093/molbev/msaa015. 32011700 PMC7182206

[13] Kolde R. 2019. pheatmap: pretty heatmaps. R package version 1.0.12. https://CRAN.R-project.org/package=pheatmap.

